# Radiation: Any Dose Is Too High

**DOI:** 10.1289/ehp.113-a735a

**Published:** 2005-11

**Authors:** Sarah Todd Davidson

Any exposure to radiation may cause cell damage that could lead to cancer, according to a June 2005 report from the National Research Council. The risk noted by the report, though small, is a third higher than the risk of 8.46 cancers per 10,000 people exposed to 1 rem (or 10 millisieverts [mSv]) currently used by U.S. regulators. The report contradicts critics who believe there is a threshold below which radiation is harmless; it also fails to support those who say low doses of radiation cause greater health damage per unit dose than high levels.

The seventh Biological Effects of Ionizing Radiation (BEIR) report, sponsored by several federal agencies, assessed and updated the health risks from low linear energy transfer (low-LET) radiation, which deposits little energy in a cell and thus tends to cause little damage. The last BEIR report that addressed these health risks was published in 1990.

Richard Monson, a professor of epidemiology at the Harvard School of Public Health and chair of the group that conducted the study, says, “We judged that the most reasonable shape is a line through the origin.” Simply put, this means any low-LET ionizing radiation may increase the risk of a cell becoming cancerous—there is no threshold below which there is no risk—and as exposure increases, so does the health risk. Researchers refer to this straight line as the linear-no-threshold model.

Less than 20% of people’s low-level radiation exposure comes from anthropogenic sources. The Earth and cosmic sources emit the remainder. Nearly 80% of human-induced exposure comes from medical procedures, about 15% from products like tobacco and building materials, and around 5% from exposure at work.

For the purpose of the BEIR VII report, the authoring committee defined low-LET radiation as levels up to about 100 mSv. For comparison, a chest X ray averages around 0.1 mSv. The committee concluded it’s likely that about 1 out of 100 people would develop a tumor or leukemia from exposure to 100 mSv above background. Of that same 100 people, experts would expect 42 to develop cancers for other reasons, but at the press conference marking the release of the report, the committee said it did not fully exclude the possibility of some radiation exposure being a factor in those cases.

The BEIR VII report employed statistical data to draw its conclusions and reviewed studies of people exposed at work and in medical settings. It also relied heavily on data from the Japanese atomic bomb survivors.

As these survivors age, more is revealed about the relationship between radiation exposure and eventual health outcomes. Investigators have also improved their estimate of the levels of exposure this population received. But critics question the heavy reliance on the Japanese survivors because of the “healthy survivor” effect—those who survived the bombing might have been hardier than those who died early on, potentially skewing the results.

Many researchers say the latest report helps reaffirm the general accuracy of federal standards in place for limiting health risks from low-level radiation. “We believe the data are more convincing than fifteen years ago and show that the radiation protection standards we use are reasonable,” says Monson.

Mike Boyd, a health physicist who works on setting and updating those standards for the Environmental Protection Agency, concurs. “I don’t think we’ll be changing any federal standards,” he says. “I’m not willing to say there will be no impact. This report will go into our estimation of risk and could lead to refinements, but generally standards should stay the same.”

Although most scientists agree the report incorporated the majority of pertinent data up through 2003, information about low-LET radiation continues to emerge. One hypothesis under investigation, says biologist Andrew Wyrobek of Lawrence Livermore National Laboratory, is the possible adaptive response cells developed over eons of natural exposure. Other hypotheses include genetic instability (the idea that some cells already have genetic mutations and are thus more prone to becoming cancerous, given the incentive) and the “bystander effect” (in which cells respond adversely to nearby irradiation although they themselves weren’t hit directly). These concepts were among those reviewed for the BEIR VII report but were not incorporated into the risk estimates.

Most experts agree that the BEIR VII report won’t be the last in the series. “Right now there is just a lot we don’t know about how cells react to very low doses of radiation,” says Wyrobek. “But with multiple exposures from more and more people undergoing medical diagnostics in the low-dose range, and increased amounts of radioactive waste, it’s important to understand these ranges better.” Says Boyd, “I will be excited to see some future academy report after we find out more about how radiation affects cells at very low doses.”

